# Alec J. Coppen—A pioneering psychiatrist who discovered the pivotal role of serotonin in the pathogenesis of depression as well as the antisuicidal effect of lithium

**DOI:** 10.1186/s40345-019-0150-3

**Published:** 2019-07-02

**Authors:** Bruno Müller-Oerlinghausen, Mohammed T. Abou-Saleh

**Affiliations:** 1 Charité Universitätsmedizin Berlin, Berlin, Germany; 2 St George’s University of London, London, UK

## Alec J. Coppen, born 1923, died on 15 March 2019

Alec Coppen, a Honorary Fellow of the Royal College of Psychiatrists has acted as director of the Medical Research Council Unit for Neuropsychiatric Research in Epsom England until 1988. Among his international colleagues he was always highly respected as a pioneering psychiatrist and psychopharmacologist credited with the discovery of the role of serotonin in the pathogenesis of depression. His seminal paper in the Lancet 1963 showed that tryptophan markedly potentiated the antidepressant action of MAOIs. He pursued the serotonin theory conducting numerous studies including studies of serotonin metabolites in post-mortem brains of depressed suicides that culminated in his 1967 paper in the British Journal of Psychiatry on the biochemistry of affective disorders that became a citation classic and made him a much honoured member of the international group of psychopharmacologists and biological psychiatrists, the CINP (Collegium Internationale Psychopharmacologicum). He acted as its president for the 17th annual CINP meeting in Kyoto in the year 1990. It is fair to state that the development of SSRI antidepressants have one origin in his work. He has published more than 500 papers and seven books on various studies of the biology of affective disorders. His other major contribution to the psychopharmacology of mood disorders was the establishment of the Lithium Clinic at West Park Hospital (in a chapter for a volume on the history of CINP he wrote: “One lesson we learned is that simply prescribing treatment is not effective”). He conducted the first placebo controlled trial of the effectiveness of lithium salts in the prophylaxis of recurrent mood disorders culminating in the discovery of lithium’s unique anti-suicidal effects concurrently with two German research groups in Berlin and Dresden. He conducted a trial of the optimum dosage of lithium establishing its minimum effective dose for prophylaxis. He studied its adverse effects on renal and thyroid functions to establish standards for safe medical practice. His last paper was a review of the effectiveness of lithium in the long-term treatment of unipolar (recurrent) depression in 2017. Alec often talked about the many letters he received from patients and their families thanking him for his good care years after he retired.

Alec had been trained in psychiatry at the Maudsley Hospital where he started his research work in biological psychiatry and attained the MD in 1958. He established the MRC Neuropsychiatry Unit at West Park Hospital in 1959 which he directed until he retired in 1988.

Alec Coppen received many honours such as the prestigious Anna Monica Prize in Biological Psychiatry Research in 1969. He was founding member of the British Association for Psychopharmacology (BAP) and was elected President 1976–1978. He was President of the Collegium Internationale Neuro-Psychopharmacologicum (CINP) 1988–1990 and was awarded the Honorary Fellowship of the Royal College of Psychiatrists in 1995. The BAP awarded him the first Gold Medal for lifetime achievement in psychopharmacology in 1998 and he was given the Pioneer of Psychopharmacology Award by the CINP in year 2000.

Alec was well read, highly cultured and enjoyed opera and theatre. In the retirement he achieved his aim of seeing all 37 Shakespeare plays performed.

He leaves his son, Michael and his grandchildren Daniel and Victoria.

